# A Dietary Model of Partial Meat Replacement with Walnuts Demonstrates Changes in the Nutrient Profile and Quality of the United States Population’s Diet

**DOI:** 10.3390/nu15214518

**Published:** 2023-10-25

**Authors:** Lisa A. Spence, Beate Henschel, Rui Li, Carmen D. Tekwe, Krisha Thiagarajah

**Affiliations:** 1 Department of Applied Health Science, School of Public Health-Bloomington, Indiana University, Bloomington, IN 47405, USA; lisspenc@iu.edu; 2 Department of Epidemiology and Biostatistics, School of Public Health-Bloomington, Indiana University, Bloomington, IN 47405, USA; bhensche@indiana.edu (B.H.); ctekwe@iu.edu (C.D.T.); 3 Department of Cardiology, Peking University Third Hospital, Beijing 100191, China; rl14@iu.edu

**Keywords:** walnuts, meat, NHANES

## Abstract

The purpose of the study is to assess the impact of partial meat replacement with walnuts using a dose–escalation approach on nutrient intake and diet quality in the usual US diet. Food modeling was implemented using the nationally representative 2015–2018 National Health and Examination Survey (NHANES), with a focus on non-nut consumers, which included 2707 children and adolescents and 5190 adults. Walnuts replaced meat in a dose-escalating manner (0.5, 1, 1.5, and 2 oz walnuts per day replaced 1, 2, 3, and 4 oz meat, respectively). Diet quality was estimated using the population ratio method of the 2015 Healthy Eating Index. The usual intake of nutrients was estimated using the National Cancer Institute method. Significant differences were determined using non-overlapping 95% confidence intervals. The partial replacement of meat with walnuts demonstrated significant increases in the mean intake of fiber, magnesium, and omega-3 fatty acids and significant decreases in cholesterol and vitamin B12 in the modeled diets for children, adolescents, and adults. Additionally, the partial replacement of meat with walnuts improved overall diet quality. Walnut consumption at 1–2 oz as a replacement for some meat may improve nutrient intake and diet quality across age groups.

## 1. Introduction

The current Dietary Guidelines for Americans (DGAs) report [1] indicates that the inadequate intake of nutrient-dense foods has resulted in the under-consumption of some nutrients. Consumers can come closer to meeting nutrient recommendations by shifting their eating patterns to include more vegetables, fruits, whole grains, nuts and seeds, and dairy. Shifting to more plant-based diet patterns in particular may provide health benefits.

Walnuts are high in fiber, potassium, calcium, magnesium, folate, vitamin E, phytosterols, polyphenols, and polyunsaturated fatty acids (PUFAs) [2], especially alpha-Linolenic acid (ALA) and linoleic acid (LA) [3]. Replacing some animal protein in one’s diet with walnuts, a plant protein, could increase the intake of several under-consumed nutrients and PUFAs while decreasing saturated fat intake, thus providing health benefits. Observational studies and randomized controlled trials support the association between walnut consumption and cardiovascular health [4,5,6,7,8]. Other observational studies have reported reduced incidences of diabetes with walnut consumption [9,10]. The consumption of walnuts may also benefit cognitive function as several components of walnuts have antioxidant and anti-inflammatory effects that may play an important role in oxidative stress and neuroinflammation [11,12].

The current study modeled the effects of a dose-escalated replacement of meat with walnuts in the usual US dietary intake pattern (0.5, 1, 1.5, and 2 oz walnuts per day replaced 1, 2, 3, and 4 oz meat because 0.5 oz nuts and 1 oz meat are considered as 1 oz equivalent [eq.] from the USDA Protein Foods Group). The researchers hypothesized that the partial replacement of meat with walnuts would improve diet quality and the intake of fiber, omega-3 PUFA, potassium, calcium, magnesium, folate, and vitamin E, and would reduce saturated fat, cholesterol, and sodium intake. This partial replacement of meat with walnuts in a dose–escalation manner was expected to lead to a favorable shift in nutrient intake to better align with dietary recommendations and increase the percentage of individuals who meet nutrient recommendations among each age–sex-stratified subpopulation. The researchers further hypothesized that replacing some meat with walnuts could improve the intake of nutrients being consumed below the recommended levels while not significantly impacting the ability to meet the recommendation for iron and vitamin B12 intake.

## 2. Materials and Methods

### 2.1. The Study Population

**Figure 1:**
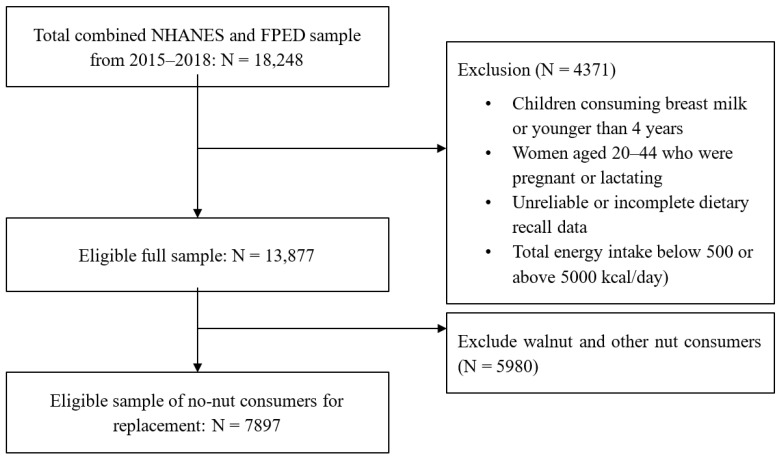
Flowchart for the study sample.

The National Health and Nutrition Examination Survey (NHANES) is a nationally representative, cross-sectional survey of the US population. NHANES data are collected by the National Center for Health Statistics (NCHSs) of the Centers for Disease Control and Prevention. Written informed consent was obtained from the participants or their proxies, and the survey protocol was approved by the Research Ethics Review Board at the NCHS. Dietary data were obtained from NHANES cycles: 2015–2016 and 2017–2018. Figure 1 illustrates this study’s population selection process. Children consuming breast milk or those younger than 4 years, women aged 20–44 years who were pregnant or lactating, respondents with unreliable food recall data, individuals reporting below 500 or above >5000 kcal/d [13], and respondents with missing information on animal protein intake (meats, cured meat, organ meat, poultry, seafood, or eggs) in the Food Patterns Equivalent Database (FPED) were excluded from our study. Prior studies indicated that tree nut consumers report better dietary quality and improved nutrient intake than no-nut consumers [14,15]. Therefore, we focused on no-nut consumers, excluding nut consumers from the analyses. The sample of no-nut consumers included 2707 children and adolescents (aged 4–18 years) and 5190 adults (19 years and older), as presented in Table 1. We divided the sample into age–gender groups; boys 4–8 years, 9–13 years, 14–18 years; girls 4–8 years, 9–13 years, 14–18 years; men 19–50 years, 51–70 years; women 19–50 years, 51–70 years; adults 71+ years.

### 2.2. Dietary Intake Measures

What We Eat in America (WWEIA) is the dietary intake interview component of the NHANES. We examined data from two non-consecutive 24 h dietary recalls, of which the first recall was completed in person at the mobile examination center with a trained interviewer, and the second was completed over the phone 3–10 days later. Detailed descriptions of the interview methods can be found in the NHANES Dietary Interviews Procedure Manual [16]. The present study included only the dietary recall data considered complete and reliable by the United States Department of Agriculture (USDA) Food Surveys Research Group. In our study sample of no-nut consumers, 79% completed both dietary recalls.

### 2.3. Food Composition Data

We obtained nutrient data from the Food and Nutrient Database for Dietary Studies (FNDDSs) and data on food consumption by food groups, such as protein foods, from the FPED. Both the FNDDSs and the FPED were mapped to the specific cycles of the NHANES data: e.g., the 2015–2016 NHANES data were analyzed with the FNDDSs 2015–2016 [17] and the FPED 2015–2016 [18], and likewise for 2017–2018 [19,20].

### 2.4. Walnut Consumption Classification

We used the FNDDSs ingredient list to identify food items that contained walnuts (ingredient codes 12155 “Nuts, walnuts, English” and 12154 “Nuts, walnuts, black, dried”) and identified NHANES respondents who consumed walnuts using the food codes for those items. Using the FPED, we identified other nut consumers who reported non-zero amounts for the component “Nuts and Seeds (Peanuts, tree nuts, and seeds (ounce equivalent); excludes coconut)” and were not already counted as walnut consumers. All remaining respondents were considered no-nut consumers. Table 1 provides the demographics of the no-nut consumers included in the modeling study.

### 2.5. Modeling

We modeled the nutritional outcomes of partial meat replacement with walnuts using a dose–escalation approach. According to the FPED documentation, the Protein Foods Groups consider 1 oz meat and 0.5 oz nuts to be 1 oz eqs. (p. 49, [20]). Therefore, we modeled the replacement of 1, 2, 3, and 4 oz meat with 0.5, 1, 1.5, and 2 oz walnuts. Meat is defined as the sum of the oz eqs. of the FPED categories of meat (beef, veal, pork, lamb, and game meat), cured meat (frankfurters, sausages, corned beef, cured ham, and luncheon meat that are made from beef, pork, or poultry), and organ meat (from beef, veal, pork, lamb, game, and poultry). The primary outcome measures were changes in the intake of potassium, dietary fiber, and magnesium—nutrients of public health concern identified by the current DGA—and iron, vitamin B12, saturated fat, and cholesterol, of which meat is a significant source [21].

According to the USDA, 1 oz of walnuts contains 185 kcal, 4.32 g protein, 1.74 g saturated fat, 2.57 g omega-3 PUFA (ALA), 10.8 g omega-6 PUFA (Linoleic acid), 27.8 mg calcium, 0.825 mg iron, 44.8 mg magnesium, 125 mg potassium 0.451 mg copper, 0.876 mg zinc, 27.8 µg dietary folate equivalents (DFEs) folate, 0.198 mg vitamin E, and 1.9 g fiber [22]. One ounce of meat (includes meat, cured meat, and organ meat but excludes poultry) contains 49 kcal, 7.1 g protein, 0.76 g saturated fat, 20 mg cholesterol, 0.01 g omega-3 PUFA (ALA), 2 mg calcium, 0.5 mg iron, 6 mg magnesium, 93 mg potassium, 1.2 mg zinc, and 0.6 µg Vitamin B12 [23].

We used the Healthy Eating Index (HEI-2015) as a composite measure of diet quality. The HEI-2015 is based on at total of 13 components, 9 that are encouraged including total fruit, whole fruit, total vegetables, greens and beans, whole grains, dairy, total protein, protein from plant, and seafood sources, and the fatty acid ratio (favoring a higher ratio of monounsaturated fatty acids and PUFAs to saturated fatty acids), and 4 that are discouraged, including refined grains, saturated fat, added sugars, and sodium. HEI scores range from 0 to 100, with higher scores indicating a better alignment with DGA and, therefore, a better diet quality. The HEI-2015 algorithm has been described previously [24]. A version of the HEI corresponding to the current DGA was not yet available upon analysis, but the nutrients and food groups that should be encouraged or limited are generally comparable between the 2020 and 2025 (current) and 2015 and 2020 DGA [1,24].

### 2.6. Analyses

We used the National Cancer Institute (NCI) method [25,26] to estimate the usual dietary intake of selected nutrients in the participants’ current diets, and this estimation was repeated after modeling the replacement of 1, 2, 3, and 4 oz meat with 0.5, 1, 1.5, and 2 oz walnuts, respectively. We used balanced repeated replication weights to account for the complex survey design used by NHANES [27]. We used the Mixtran (version 2.1), Distrib (version 2.1), and BRR_PValue_CI (version 1.0) macros provided by the NCI to run our analyses. Covariates in the NCI method included the sequence of the participant’s intake (day 1 or 2), age (7 categories), gender (male/female), race (4 categories), energy intake (kcal), and an indicator for weekday consumption as Monday–Thursday and weekend consumption as Friday–Sunday.

We calculated the mean and corresponding 95% confidence intervals (CIs) for each nutrient of interest and age–gender subgroup and determined the percentages of the population with intakes below the estimated adequate requirement (EAR) for magnesium, vitamin B12, and iron. We also examined the percentages of the population with intakes above the recommended adequate intake (AI) for potassium, fiber, cholesterol, and ALA, as recommended by published Dietary Reference Intakes [28].

The HEI-2015 score was estimated using the population ratio method described previously [29] with data from the first 24 h recall (day 1). We adapted the SAS macro provided by the NCI (hei2015.score.macro.sas) [26] for the 2015–2016 and 2017–2018 NHANES and FPED cycles used in this study. We modeled HEI scores based on reported intake, adding the nutrient contents of walnuts and subtracting the nutrient contents of meat in the dose-escalating manner described above.

The following values were used for the addition of 1 oz walnuts: 2.53 g monounsaturated fatty acids, 13.4 g PUFAs, 1.74 g saturated fats, 185 calories, 2 oz eq. total protein, 2 oz eq. seafood and plant proteins, and 0.567 mg sodium. Similarly, we used the following values to subtract 1 oz eq. meat: 0.87 g monounsaturated fatty acids, 0.12 g PUFAs, 0.76 g saturated fats, 49 calories, 1 oz eq. total protein, 1 oz eq. seafood and plant proteins, and 127 mg sodium.

### 2.7. Cost Analysis

We followed a multi-step process to assess the financial influence on the consumers’ spending with partial replacement of meat with walnuts. We first obtained food prices from the Center for Nutrition Policy and Promotion Food Prices Database for 2003–2004 [30], which includes the average prices of approximately 4600 food items (excluding alcoholic beverages) reported by participants of the 2003–2004 NHANES cycle [31]. In the second step, we merged food codes and food categories from the 2015 to 2016 and 2017 to 2018 cycles of the WWEIA database and FNDDSs [32] into the Food Prices Database to prepare the dataset for inflation adjustment. However, approximately 5000 food items reported by participants in the 2015–2016 and 2017–2018 NHANES cycles were not in the 2003–2004 Food Prices Database. Therefore, in the third step, we assigned prices to those new food items using prices from similar food items.

In the fourth step, we adjusted the 2003–2004 food prices for inflation using the consumer price index (obtained from the USDA Economic Research Service [33]) by food categories. In the fifth step, we calculated the daily cost of all food/meat items by multiplying the inflation-adjusted prices by the consumed amount of food/meat items from day 1 of the food recall. Meat/food items were classified using the WWEIA food categories “Protein Foods–Meats” and “Protein Foods–Cured Meats/Poultry”.

In step 6, we calculated the daily cost of the partial replacement of meat with walnuts for the four replacement levels detailed above (0.5 oz walnuts replacing 1 oz meat, …, 2 oz walnuts replacing 4 oz meat). If an NHANES participant reported meat consumption of less than 4 oz, we only modeled the replacement of meat up to the reported level. Similarly, if a participant reported no meat consumption, we held their daily food expenses constant. Lastly, we obtained the average daily expenses for specific age (4–18 years, 19–50 years, 51–70 years, and 71 years and older) and gender groups for the current diet and the four modeled diets considering the NHANES survey weights.

The results are presented by age and gender subgroups. Significant differences between nutrient intakes (including % below EAR and % above adequate intake) and HEI scores for the current and modeled intakes were determined using non-overlapping 95% CIs. We used version 9.4 SAS software (SAS Institute Inc., Cary, NC, USA) for all data analyses with an alpha level of 0.05.

## 3. Results

In our sample of no-nut consumers, approximately 25% were children or adolescents, 44% were between 19 and 50 years old, and 31% were 51 years or older. Fifty-two percent of the sample was male. The majority were non-Hispanic white (52%), had an annual household income between USD 20,000 and 75,000 (50%), and had at least some college education (42%).

Table 2 presents the total mean protein food intake and the distribution of type of protein food intake among children and adults. Over 90% of the protein food intake expressed in oz equivalents was from animal food (meat, poultry, seafood, and eggs), and, of this, over 50% was from meat. In this study, meat includes meat, cured meat, and organ meat but excludes poultry. Moreover, boys/men consumed more protein than girls/women.

**Figure 2:**
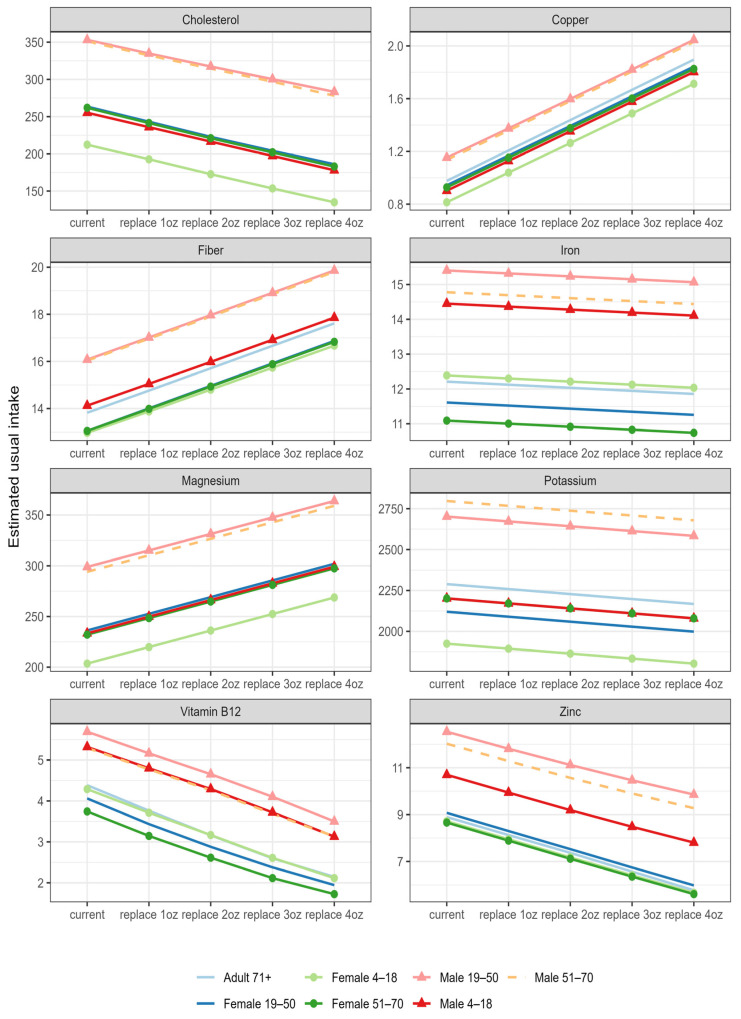
Nutrient intake for usual diet (current) and modeled diet when walnuts replaced meat in a dose-escalating manner (i.e., 0.5, 1, 1.5, and 2 oz walnuts per day replaced 1, 2, 3, and 4 oz meat per day, respectively).

The results of the food modeling study suggest that replacing up to 4 ounces of meat with walnuts may improve the intake of several nutrients inadequately consumed by the US population. For all the age and gender groups, the partial replacement of meat with walnuts in the modeled diet resulted in a greater intake of magnesium, copper, and fiber and a lower intake of cholesterol and vitamin B12. No significant changes were observed in iron and potassium intake (Figure 2). Similar patterns were observed in all gender and age groups.

### 3.1. Change in Percent of Population Meeting Recommendations

Replacing meat with walnuts in a dose-escalating manner (replacing 1, 2, 3, and 4 oz meat with 0.5, 1, 1.5, and 2 oz walnuts, respectively) resulted in a significant decline in protein intake in all four models for children and adults (see Supplemental Table S5). The Acceptable Macronutrients Distribution Range (AMDR) for protein is 10–35% of energy intake. The current dietary intake meets the AMDR recommendation for protein for all age and gender groups, ranging from 14% to 16% of energy intake as protein. When replacing meat (1, 2, 3, 4 oz) with walnuts (0.5, 1, 1.5, and 2 oz, respectively), the AMDR recommendation for protein was met for all age and gender categories, but only up to 1 oz walnuts.

When replacing meat with walnuts in a dose-escalating manner, intake of magnesium significantly increased in all four models for children (4–18 years) and adults (Figure 2). Replacing 2 oz meat with 1 oz walnuts was enough to significantly improve magnesium intake in all age and gender groups. A similar observation was noted for fiber intake. Moreover, replacing 4 oz meat with 2 oz walnuts significantly improved potassium intake in children and adults compared with the usual diet, but the same effect was not observed for the other modeled doses.

Vitamin B12 intake significantly decreased in all four models for children (4–18 years) and adults (Figure 2). Furthermore, a minimum of 1 oz walnuts (2 oz meat eq.) significantly decreased vitamin B12 intake in the modeled diet for all age and gender groups. Replacing meat with walnuts also led to a significant decline in zinc intake in children and adults but improved copper intake, especially with a replacement of 1 oz or more of walnuts (Figure 2).

### 3.2. Changes in Dietary Fat Intake

Replacing 1 oz meat with 0.5 oz walnuts allowed for the omega-3 fatty acids recommendation to be met and significantly increased PUFA and linoleic acid intake in the modeled diet for all age and gender categories.

### 3.3. Changes in Diet Quality

We examined the HEI-2015 to measure the influence of the replacement models on diet quality (Table 3). Based on current diets, the population mean HEI-2015 value was 49.1 (95% CI 47.94, 50.37) for children and adolescents (4–18 years) and 52.4 (95% CI 50.99, 53.79) for adults (19 years and older). Replacing 1, 2, 3, and 4 oz meat with 0.5, 1, 1.5, and 2 oz walnuts, respectively, significantly improved diet quality for children (4–18 years) and adults (19 years and older) (HEI-2015 scores for children: 55.0, 57.6, 60.2, 61.8; for adults: 56.4, 59.0, 61.5, 62.4, respectively). Further, adding 1.5–2 oz walnuts to the diet significantly improved diet quality in all gender and age subcategories. When evaluating individual HEI-2015 components, values for seafood and plant protein and the fatty acid ratio improved and contributed most to the improved diet quality.

### 3.4. Changes in Cost with Replacement

The estimated cost per ounce of meat was USD 0.38 in both 2015–2016 and 2017–2018, and the cost per ounce of walnuts was USD 0.26 in 2015–2016 and USD 0.27 in 2017–2018. Although daily expenses for food decreased by USD 0.08–0.13 (1 oz replacement, decrease of 1.4–2.1%), USD 0.13–0.22 (2 oz replacement, decrease of 2.5–3.7%), USD 0.16–0.32 (3 oz replacement, decrease of 3.3–4.9%), and USD 0.19–0.39 (4 oz replacement, decrease of 3.8–5.6%), these changes were not significant when compared with the current daily expenses.

## 4. Discussion

Over 90% of the protein food intake in the current study came from animal sources, and of animal-sourced protein food, more than 50% came from meat. The saturated fat intake for all age and gender categories was above 10% of the total calories of the current dietary intake. The DGA recommends increasing consumption of unsaturated fatty acids and limiting saturated fat intake. We hypothesized that partially replacing meat with walnuts might reduce the intake of saturated fat and cholesterol. However, this modeling study demonstrated that partially replacing meat with walnuts did not significantly reduce the saturated fat intake, although a trend toward decreased intake was observed. Compared with meat, nuts generally have an intermediate amount of saturated fat, whereas other plant-based proteins have lower levels of saturated fat [34], which could potentially reduce saturated fat intake. We also observed significant increases in polyunsaturated fat intake along with reductions in cholesterol intake in the modeled diet of partial replacement of meat with walnuts.

Increasing PUFA intake by partially replacing meat with walnuts, as examined in this modeling study, may provide health benefits. Studies indicate that the abundance of omega-3 and omega-6 fatty acids in walnuts may contribute to their cardioprotective properties [5]. Other studies indicate that intakes of red and processed meat are associated with modest increases in total mortality, cardiovascular disease mortality [35,36], and cancer mortality [36]. A modeling study demonstrated that replacing red and processed meat with other protein sources may reduce the incidence of type 2 diabetes [37]. Moreover, red meat consumption may increase the risk of frailty in older women [38]. Despite the health concerns associated with processed meat, the processed meat intake among adults 20 years and older has not changed since 1999, although there has been a decline in unprocessed red meat intake [39]. The 2020–2025 DGA cites a need to improve the US diet, further noting that small shifts in eating patterns can make a considerable difference in overall diet quality [1].

Some nutrient concerns with reducing meat intake include the potential for reduced iron intake and vitamin B12 deficiency [40]. We hypothesized that replacing some meat with walnuts could increase the intake of nutrients such as magnesium, potassium, and fiber to meet the recommended intake levels while not significantly impacting the intake of iron and vitamin B12. The current modeling study found no significant changes in iron intake when meat was partially replaced with walnuts in a dose-escalating manner. A similar outcome was observed in a modeling study conducted with young, Dutch, female participants [41]. In this modeling study, replacing at least 2 oz of meat with walnuts showed a significant reduction in mean vitamin B12 intake for all age and gender groups. However, all age and gender groups maintained a mean vitamin B12 intake above the recommended daily allowance when replacing 2–3 oz of meat with walnuts. Nevertheless, the percentage of participants below the EAR for vitamin B12 increased with meat replacement. A similar observation was noted for zinc. Therefore, it is important to monitor vitamin B12 and zinc intake in diets that may replace meat with walnuts. This modeling study observed a significant increase in the mean intake of magnesium, copper, and fiber for all age and gender groups when replacing at least 2 oz of meat with walnuts. A similar result was found in another study, which modeled a reference omnivore diet using NHANES 2017–2018 data and compared it with diets that substituted animal products in the reference diet with either traditional or novel plant-based foods to create flexitarian, vegetarian, and vegan diets matched for calories and macronutrients [42].

Studies have shown that limiting meat intake may improve diet quality [43,44]. In this modeling study, replacing some meat with walnuts significantly improved diet quality. Moreover, our cost analysis indicates that replacing meat with walnuts does not increase the cost of food in the diet; instead, there is a declining trend in cost, although it is not significant compared with current food cost.

The strengths of this study include its use of data from the nationally representative NHANES survey of 2015–2018 and its consideration of multiple doses in a dose-escalating replacement of meat with walnuts. Furthermore, the study assessed the range of meat intake that could be replaced with walnuts without compromising diet quality and nutrient intake. A limitation of this study is its use of 24 h dietary recalls, which might be subject to error when assessing nutrient intake. The cross-sectional survey design of NHANES data allowed us to examine population level associations, but it prevents us from assessing causality.

## 5. Conclusions

The results of this modeling study reveal that the partial replacement of meat with 1–1.5 oz walnuts may improve the dietary intake of some nutrients, such as dietary fiber, magnesium, and PUFA, including omega-3 fatty acids, and may decrease the intake of cholesterol among the US population. However, it is not likely to change saturated fat intake. The partial replacement of meat with walnuts enhanced overall diet quality, as assessed by HEI. Individuals replacing meat with walnuts in their diet should be cautious about the potential risk of zinc and vitamin B12 deficiencies.

## Figures and Tables

**Table 1:** Demographic characteristics of no-nut consumers from the NHANES, 2015–2018.

	No-Nut Consumers (*n* = 7.897)	
	*n*	Weighted Statistics ^1^
**Age group, %**		
4 to 8 years	850	7.36 ± 0.30
9 to 13 years	949	8.35 ± 0.35
14 to 18 years	908	9.03 ± 0.48
19 to 50 years	2732	44.49 ± 1.06
51 to 70 years	1745	22.79 ± 0.91
71 years and older	713	7.98 ± 0.57
**Gender, %**		
Men	4049	52.61 ± 0.65
Women	3848	47.39 ± 0.65
**Race/Hispanic origin, %**		
Mexican American and Other Hispanic	2447	22.03 ± 2.29
Non-Hispanic White	2194	51.96 ± 2.76
Non-Hispanic Black	2047	15.54 ± 1.72
Other Race	1209	10.47 ± 0.99
**Annual household income, %**		
less than USD 20,000	1673	17.89 ± 1.29
USD 20,000 to 75,000	3877	49.52 ± 1.77
USD 75,000 to 99,999	676	11.11 ± 0.68
over USD 100,000	1077	21.48 ± 1.55
**Ratio of family income to poverty**	7016	2.51 ± 0.06
**Education, %**		
Less than 9th grade	2359	20.76 ± 0.65
9th–11th grade (Incl. 12th grade with no diploma)	1249	13.81 ± 0.69
High school graduate/GED or equivalent	1472	24.06 ± 0.76
Some college or associate degree	1620	25.73 ± 0.88
College degree or above	871	15.64 ± 1.13

^1^ Data are presented as weighted mean ± standard error (SE) or weighted percentage ± SE.

**Table 2:** Usual intake of protein foods (NHANES 2015–2016 and 2017–2018) among no-nut consumers.

Age–Gender Group ^1^	Total Intake of Protein Foods	Meats ^2^	Poultry	Seafood	Eggs	Legumes	Soy
	oz Equivalents (%)						
Boys	4.86	2.47 (50.9)	1.43 (29.4)	0.22 (4.5)	0.38 (7.8)	0.32 (6.5)	0.05 (1.0)
Girls	3.81	1.71 (44.9)	1.21 (31.7)	0.21 (5.5)	0.37 (9.8)	0.26 (6.9)	0.05 (1.3)
Children	4.36	2.11 (48.4)	1.32 (30.3)	0.21 (4.9)	0.38 (8.6)	0.29 (6.7)	0.05 (1.1)
Men	6.97	3.44 (49.4)	1.71 (24.5)	0.65 (9.4)	0.60 (8.6)	0.53 (7.6)	0.04 (0.6)
Women	5.05	2.20 (43.6)	1.32 (26.2)	0.58 (11.5)	0.53 (10.5)	0.38 (7.4)	0.04 (0.8)
Adults	6.06	2.85 (47.1)	1.53 (25.2)	0.62 (10.2)	0.57 (9.3)	0.46 (7.5)	0.04 (0.6)

^1^ Boys, girls, and children 4 to 18 years old. Men, women, and adults 19 years and older. ^2^ Meats include meats, cured meat, and organ meat.

**Table 3:** HEI total score for usual diet (current) and modeled diet when walnuts replaced meat in a dose-escalating manner (i.e., 0.5, 1, 1.5, and 2 oz walnuts per day replace 1, 2, 3, and 4 oz meat per day, respectively).

Age–Gender Group	HEI Total Score Replace Meat ^1^				
	Current	Replacement 1 oz	Replacement 2 oz	Replacement 3 oz	Replacement 4 oz
Male 4–8	51.4 (48.9–53.8)	57.4 (54.9–59.8) *	60.1 (57.7–62.6) *	62.7 (60.2–65.2) *	64.7 (62.4–67.0) *
Male 9–13	46.8 (44.3–49.3)	52.4 (49.8–54.8) *	55.3 (52.8–57.7) *	57.7 (55.3–60.0) *	59.8 (57.4–62.1) *
Male 14–18	44.6 (42.4–46.8)	49.8 (47.6–51.9) *	52.6 (50.5–54.8) *	54.9 (52.7–57.1) *	57.1 (54.9–59.2) *
Male 4–18	47.3 (45.7–48.9)	52.8 (51.1–54.5) *	55.6 (54.0–57.2) *	58.0 (56.3–59.7) *	60.2 (58.6–61.7) *
Female 4–8	54.1 (52.2–56.0)	60.8 (58.8–62.7) *	63.8 (61.8–65.8) *	66.8 (64.7–68.7) *	67.6 (65.8–69.5) *
Female 9–13	51.6 (49.1–54.2)	57.3 (54.9–59.7) *	59.9 (57.6–62.3) *	62.5 (60.1–64.8) *	63.4 (61.4–65.4) *
Female 14–18	48.7 (45.9–51.5)	54.4 (51.8–57.1) *	57.3 (54.7–60.1) *	59.6 (57.1–62.1) *	60.2 (57.9–62.7) *
Female 4–18	51.5 (50.1–53.1)	57.4 (56.1–58.8) *	60.2 (58.9–61.6) *	62.9 (61.6–64.2) *	63.6 (62.4–64.8) *
Child 4–18	49.1 (47.9–50.4)	55.0 (53.8–56.2) *	57.6 (56.5–58.8) *	60.2 (59.0–61.3) *	61.8 (60.8–62.8) *
Male 19–50	49.7 (47.9–51.7)	53.7 (52.1–55.3) *	55.9 (54.3–57.5) *	58.1 (56.5–59.7) *	59.9 (58.4–61.3) *
Female 19–50	50.7 (48.4–53.0)	55.3 (53.1–57.5) *	58.1 (56.0–60.3) *	60.7 (58.7–62.6) *	61.4 (59.5–63.2) *
Adult 19–50	50.1 (48.4–51.9)	54.3 (52.8–55.8) *	56.8 (55.3–58.3) *	59.2 (57.7–60.7) *	60.5 (59.2–61.8) *
Male 51–70	54.0 (51.1–57.0)	57.3 (54.6–60.0)	59.5 (56.8–62.3)	61.8 (59.0–64.6) *	63.2 (60.8–65.6) *
Female 51–70	56.4 (54.7–58.1)	60.7 (58.9–62.5) *	63.7 (61.9–65.5) *	65.6 (63.9–67.2) *	66.2 (64.5–67.8) *
Adult 51–70	55.0 (53.0–56.9)	58.7 (56.7–60.7)	61.3 (59.3–63.3) *	63.7 (61.8–65.6) *	64.4 (62.7–66.2) *
Adult 71+	59.6 (56.4–62.7)	63.6 (60.8–66.3)	66.5 (63.7–69.2) *	68.7 (66.0–71.2) *	69.2 (66.6–71.7) *
Male 19–71+	51.7 (50.1–53.2)	55.4 (54.0–56.8) *	57.6 (56.2–59.0) *	59.9 (58.5–61.3) *	61.5 (60.2–62.8) *
Female 19–71+	53.5 (51.9–55.2)	58.0 (56.3–59.7) *	60.9 (59.2–62.6) *	63.2 (61.8–64.7) *	63.8 (62.4–65.3) *
Adult 19–71+	52.4 (51.0–53.8)	56.4 (55.1–57.8) *	59.0 (57.6–60.3) *	61.5 (60.1–62.8) *	62.4 (61.2–63.6) *

^1^ Meats include meats, cured meat, and organ meat. * 95% confidence intervals of HEI total score– usual diet and modeled replacement do not overlap.

## Data Availability

Data used in the manuscript are available at https://www.cdc.gov/nchs/nhanes/index.htm (accessed on 1 December 2020). Analytic code will be made available upon request.

## Supplementary Materials

The following supporting information can be downloaded at: https://www.mdpi.com/article/10.3390/nu15214518/s1, Supplemental Table S1: Percentage of individuals with nutrient intakes below the EAR ^a^ at current intake (usual diet) and modeled intake when replacing meat with equivalent^b^ amount of walnuts. Supplemental Table S2: Percentage of individuals below EAR ^a^ for magnesium and above AI ^b^ for fiber (both under-consumed nutrients) at current intake (usual diet) and modeled intake when replacing meat with equivalentc amount of walnuts. Supplemental Table S3: Percentage of individuals with nutrient intake levels above the AI ^a^ at current intake (usual diet) and modeled intake when replacing meat with equivalentb amount of walnuts. Supplemental Table S4: Percentage of individuals with nutrient intakes below the EAR ^a^ at current intake (usual diet) and modeled intake when replacing meat with equivalentb amount of walnuts. Supplemental Table S5: AMDRa of protein intake (protein as % energy) at current intake and modeled intake when replacing meat with equivalentb amount of walnuts. Supplemental Table S6: Saturated fat intake in grams and percentage of individuals with cholesterol intake levels above 300 mg at current intake (usual diet) and modeled for replacing meat with an equivalenta amount of walnuts. Supplemental Table S7: Percentage of individuals with nutrient intake levels above the AI ^a^ at current intake (usual diet) and modeled at replacing meat with an equivalentb amount of walnuts.
