# Supplementary material for: A Dietary Model of Partial Meat Replacement with Walnuts Demonstrates Changes in the Nutrient Profile and Quality of the United States Population’s Diet
Source: Nutrients. 2023 Oct 25;15(21):4518. doi: 10.3390/nu15214518 (PMC10648433; doi:10.3390/nu15214518)
Supplement: Supplementary file 1 [file nutrients-15-04518-s001.zip › nutrients-2638816-SI.pdf]

Supplemental Table S1: Percentage of individuals with nutrient intakes below the EAR[a] at current intake (usual diet) and modeled intake when replacing meat with equivalent[b] amount of walnuts

**Age-gender group**	**Vitamin B12** % below EAR (95% confidence interval)					**Iron** % below EAR (95% confidence interval)				
	Current	Replacement 1 oz eq	Replacement 2 oz eq	Replacement 3 oz eq	Replacement 4 oz eq	Current	Replacement 1 oz eq	Replacement 2 oz eq	Replacement 3 oz eq	Replacement 4 oz eq
Male 4–8	0.2 (0.0-0.4)	1.5 (0.7-2.2)*	4.3 (2.6-6.0)*	9.4 (6.6-12.1)*	16.8 (12.4-21.2)*	0.1 (-0.0-0.3)	0.2 (0.0-0.4)	0.2 (0.0-0.5)	0.3 (0.0-0.5)	0.3 (0.0-0.5)
Male 9–13	1.3 (0.6-2.0)	5.1 (3.2-7.0)*	10.9 (8.0-13.8)*	18.3 (14.4-22.3)*	26.1 (21.1-31.1)*	1.5 (0.5-2.5)	1.6 (0.6-2.7)	1.8 (0.6-2.9)	1.9 (0.7-3.2)	2.2 (0.8-3.5)
Male 14–18	5.3 (3.6-7.1)	13.2 (10.5-16.0)*	22.0 (18.5-25.5)*	30.4 (26.7-34.2)*	39.2 (35.0-43.4)*	9.5 (6.2-12.8)	9.9 (6.5-13.3)	10.4 (6.9-14.0)	10.9 (7.2-14.6)	11.5 (7.7-15.3)
Male 4–18	2.5 (1.6-3.4)	7.1 (5.5-8.6)*	13.1 (10.9-15.2)*	20.2 (17.7-22.6)*	28.2 (25.2-31.2)*	4.1 (2.6-5.6)	4.3 (2.8-5.9)	4.6 (3.0-6.2)	4.8 (3.1-6.5)	5.1 (3.4-6.8)
Female 4–8	0.6 (0.0-1.2)	3.1 (1.5-4.6)*	8.3 (5.8-10.9)*	17.7 (13.7-21.7)*	29.8 (24.8-34.9)*	0.3 (0.1-0.6)	0.4 (0.1-0.6)	0.4 (0.1-0.7)	0.5 (0.1-0.8)	0.5 (0.1-0.9)
Female 9–13	2.7 (1.5-3.9)	9.1 (6.6-11.5)*	17.8 (13.9-21.7)*	29.2 (24.1-34.2)*	40.3 (34.7-45.9)*	1.7 (0.8-2.5)	1.8 (0.9-2.7)	2.0 (1.0-3.0)	2.2 (1.1-3.2)	2.3 (1.2-3.4)
Female 14–18	12.0 (8.5-15.5)	25.9 (21.7-30.2)*	40.1 (35.4-44.9)*	52.7 (48.3-57.0)*	63.4 (59.2-67.5)*	19.3 (15.3-23.2)	20.0 (15.9-24.1)	21.0 (16.8-25.2)	21.8 (17.6-26.1)	22.7 (18.3-27.1)
Female 4–18	5.4 (3.7-7.1)	13.3 (10.9-15.7)*	22.9 (19.9-25.9)*	34.1 (30.6-37.6)*	45.4 (41.8-49.0)*	7.5 (6.0-9.0)	7.8 (6.2-9.4)	8.2 (6.6-9.9)	8.6 (6.9-10.3)	9.0 (7.3-10.7)
Child 4–18	3.9 (2.6-5.1)	10.0 (8.2-11.8)*	17.8 (15.5-20.0)*	26.8 (24.2-29.4)*	36.4 (33.5-39.2)*	5.7 (4.5-7.0)	6.0 (4.7-7.3)	6.3 (5.0-7.7)	6.6 (5.2-8.0)	7.0 (5.5-8.4)
Male 19–50	5.0 (3.5-6.5)	12.4 (9.9-15.0)*	20.5 (17.4-23.6)*	28.6 (24.9-32.2)*	37.1 (33.1-41.0)*	3.4 (2.5-4.3)	3.7 (2.7-4.6)	3.9 (2.9-4.8)	4.1 (3.1-5.1)	4.4 (3.3-5.4)
Female 19–50	13.6 (10.6-16.6)	28.1 (24.1-32.0)*	42.1 (37.4-46.8)*	54.5 (49.8-59.2)*	64.9 (60.4-69.4)*	25.2 (21.9-28.5)	26.0 (22.6-29.3)	26.8 (23.4-30.2)	27.7 (24.3-31.1)	28.6 (25.1-32.1)
Adult 19–50	8.9 (6.8-11.0)	19.5 (16.6-22.5)*	30.3 (26.9-33.8)*	40.3 (36.6-44.0)*	49.7 (46.0-53.4)*	13.3 (11.4-15.2)	13.8 (11.9-15.7)	14.3 (12.4-16.2)	14.8 (12.9-16.8)	15.4 (13.3-17.4)
Male 51–70	5.6 (3.9-7.3)	13.6 (11.1-16.1)*	22.9 (19.8-25.9)*	32.6 (28.9-36.4)*	41.9 (37.9-45.8)*	3.0 (2.1-3.9)	3.2 (2.2-4.1)	3.4 (2.4-4.3)	3.6 (2.6-4.6)	3.8 (2.8-4.8)
Female 51–70	16.8 (12.7-21.0)	33.1 (28.1-38.1)*	48.3 (43.0-53.7)*	61.6 (56.2-66.9)*	71.2 (66.1-76.3)*	3.6 (2.6-4.6)	3.9 (2.9-5.0)	4.3 (3.2-5.4)	4.6 (3.5-5.8)	5.0 (3.8-6.1)
Adult 51–70	11.0 (8.3-13.8)	23.0 (19.5-26.5)*	35.2 (31.4-39.1)*	46.6 (42.6-50.7)*	56.1 (52.2-60.0)*	3.3 (2.4-4.1)	3.5 (2.6-4.4)	3.8 (2.9-4.7)	4.1 (3.1-5.1)	4.4 (3.4-5.3)
Adult 71+	9.4 (4.3-14.4)	21.5 (13.6-29.3)	35.6 (26.2-45.0)*	49.2 (40.8-57.6)*	60.0 (51.7-68.3)*	2.3 (1.1-3.5)	2.5 (1.2-3.8)	2.7 (1.4-4.1)	3.0 (1.6-4.4)	3.2 (1.8-4.7)
Male 19–71+	5.2 (3.7-6.7)	12.9 (10.6-15.3)*	21.6 (18.8-24.4)*	30.5 (27.3-33.6)*	39.4 (36.1-42.6)*	3.2 (2.6-3.9)	3.4 (2.8-4.1)	3.7 (3.0-4.3)	3.9 (3.2-4.6)	4.1 (3.4-4.9)
Female 19–71+	14.5 (11.1-17.8)	29.6 (25.4-33.8)*	44.3 (39.8-48.9)*	57.4 (53.1-61.6)*	67.6 (63.5-71.7)*	15.6 (13.7-17.6)	16.2 (14.2-18.2)	16.9 (14.8-18.9)	17.5 (15.4-19.6)	18.1 (16.0-20.2)
Adult 19–71+	9.6 (7.3-11.9)	20.8 (17.8-23.8)*	32.4 (29.0-35.7)*	43.2 (39.8-46.5)*	52.7 (49.4-56.0)*	9.1 (8.0-10.2)	9.5 (8.4-10.6)	9.9 (8.8-11.0)	10.3 (9.2-11.5)	10.7 (9.6-11.9)

*95% confidence intervals of percentages from those below/above recommended and modeled replacement do not overlap

[a]EAR, estimated average requirement: A nutrient intake value that is estimated to meet the requirement of half the healthy individuals in a group

[b]One ounce equivalent (eq) per Protein Foods Group is 0.5 oz nuts and 1 oz meat (20, p. 49). Therefore, we modeled the replacement of 1, 2, 3, and 4 oz eqs meat with 0.5, 1, 1.5, and 2 oz walnuts.

Supplemental Table S2: Percentage of individuals below EAR[a] for magnesium and above AI[b] for fiber (both under-consumed nutrients) at current intake (usual diet) and modeled intake when replacing meat with equivalent[c] amount of walnuts

Age-gender group	**Magnesium** % below EAR (95% confidence interval)					**Fiber (g/1,000 kcal)** % above AI (95% confidence interval)				
	Current	Replacement 1 oz eq	Replacement 2 oz eq	Replacement 3 oz eq	Replacement 4 oz eq	Current	Replacement 1 oz eq	Replacement 2 oz eq	Replacement 3 oz eq	Replacement 4 oz eq
Male 4–8	4.4 (2.9-5.9)	1.8 (1.0-2.6)*	0.7 (0.3-1.0)*	0.2 (0.0-0.4)*	0.0 (0.0-0.1)*	1.0 (0.4-1.7)	1.1 (0.4-1.9)	1.3 (0.5-2.2)	1.7 (0.7-2.7)	2.1 (1.0-3.3)
Male 9–13	41.2 (35.2-47.1)	32.0 (26.2-37.9)	23.6 (18.4-28.8)*	16.5 (11.9-21.1)*	10.8 (7.2-14.5)*	0.8 (0.3-1.3)	0.9 (0.3-1.4)	1.1 (0.4-1.7)	1.3 (0.5-2.1)	1.6 (0.6-2.6)
Male 14–18	82.7 (79.6-85.9)	79.3 (75.9-82.8)	75.2 (71.4-78.9)*	70.3 (66.3-74.3)*	65.1 (60.8-69.4)*	0.4 (0.1-0.8)	0.5 (0.1-0.9)	0.6 (0.2-1.1)	0.8 (0.3-1.3)	0.9 (0.3-1.6)
Male 4–18	45.7 (43.3-48.0)	40.7 (38.3-43.0)*	36.1 (33.8-38.3)*	31.8 (29.6-34.0)*	28.0 (25.8-30.1)*	0.7 (0.2-1.2)	0.8 (0.3-1.4)	1.0 (0.4-1.6)	1.2 (0.5-1.9)	1.5 (0.7-2.4)
Female 4–8	6.3 (4.7-7.8)	2.7 (1.8-3.7)*	1.1 (0.5-1.6)*	0.4 (0.1-0.6)*	0.1 (0.0-0.2)*	2.0 (1.0-3.0)	2.3 (1.2-3.3)	2.7 (1.5-3.8)	3.2 (1.8-4.5)	3.8 (2.3-5.4)
Female 9–13	50.6 (45.2-55.9)	40.5 (35.2-45.8)	30.9 (26.0-35.8)*	21.9 (17.6-26.2)*	14.5 (11.0-18.0)*	1.1 (0.5-1.8)	1.3 (0.5-2.0)	1.6 (0.8-2.4)	1.9 (1.0-2.7)	2.2 (1.3-3.1)
Female 14–18	89.0 (85.9-92.0)	86.0 (82.7-89.4)	81.8 (78.2-85.5)*	76.8 (73.0-80.6)*	70.9 (66.9-75.0)*	0.9 (0.3-1.4)	1.0 (0.4-1.6)	1.3 (0.6-2.0)	1.6 (0.8-2.4)	2.0 (1.0-2.9)
Female 4–18	51.2 (48.3-54.1)	45.6 (42.6-48.5)	40.2 (37.2-43.1)*	35.0 (32.2-37.8)*	30.2 (27.6-32.9)*	1.3 (0.6-2.0)	1.5 (0.7-2.2)	1.8 (1.0-2.6)	2.2 (1.3-3.1)	2.6 (1.6-3.6)
Child 4–18	48.3 (46.1-50.5)	43.0 (40.9-45.1)*	38.0 (36.0-40.0)*	33.3 (31.4-35.2)*	29.0 (27.3-30.8)*	1.0 (0.4-1.6)	1.1 (0.5-1.7)	1.4 (0.7-2.0)	1.7 (0.9-2.4)	2.0 (1.1-2.9)
Male 19–50	66.0 (62.7-69.2)	60.8 (57.5-64.2)	55.3 (51.7-58.8)*	49.5 (45.8-53.2)*	43.2 (39.6-46.9)*	0.4 (0.1-0.8)	0.5 (0.1-0.8)	0.5 (0.1-1.0)	0.7 (0.2-1.2)	0.8 (0.2-1.4)
Female 19–50	65.2 (61.5-69.0)	57.8 (53.8-61.7)	49.7 (45.9-53.5)*	41.1 (37.5-44.8)*	32.3 (28.8-35.9)*	0.7 (0.2-1.2)	0.9 (0.3-1.5)	1.1 (0.5-1.8)	1.4 (0.7-2.2)	1.8 (0.9-2.7)
Adult 19–50	65.6 (62.7-68.6)	59.4 (56.4-62.5)*	52.8 (49.6-55.9)*	45.7 (42.5-48.8)*	38.3 (35.2-41.4)*	0.6 (0.1-1.0)	0.7 (0.2-1.1)	0.8 (0.3-1.3)	1.0 (0.4-1.6)	1.3 (0.6-2.0)
Male 51–70	73.0 (69.5-76.4)	68.9 (65.3-72.5)	64.1 (60.4-67.8)*	58.4 (54.7-62.1)*	52.6 (48.8-56.3)*	0.6 (0.1-1.2)	0.7 (0.1-1.3)	0.8 (0.2-1.5)	1.1 (0.3-1.9)	1.3 (0.5-2.2)
Female 51–70	72.3 (67.7-76.9)	65.5 (60.6-70.4)	57.7 (52.3-63.0)*	49.1 (43.8-54.5)*	39.8 (34.7-44.9)*	1.4 (0.6-2.1)	1.6 (0.8-2.5)	2.0 (1.1-3.0)	2.5 (1.4-3.6)	3.1 (1.8-4.4)
Adult 51–70	72.6 (70.1-75.2)	67.3 (64.5-70.0)*	61.0 (57.9-64.1)*	53.9 (50.7-57.1)*	46.4 (43.1-49.7)*	1.0 (0.3-1.6)	1.2 (0.5-1.8)	1.4 (0.6-2.2)	1.8 (0.9-2.7)	2.2 (1.2-3.2)
Adult 71+	82.8 (80.2-85.5)	77.5 (74.3-80.7)	71.2 (67.5-75.0)*	64.0 (59.9-68.0)*	55.8 (51.2-60.4)*	2.4 (1.1-3.7)	2.9 (1.5-4.4)	3.5 (1.9-5.2)	4.3 (2.3-6.2)	5.2 (3.0-7.4)
Male 19–71+	69.8 (67.5-72.2)	65.2 (62.7-67.7)	60.0 (57.3-62.6)*	54.3 (51.6-57.1)*	48.3 (45.5-51.0)*	0.6 (0.2-1.0)	0.7 (0.2-1.1)	0.8 (0.3-1.3)	1.0 (0.4-1.6)	1.2 (0.5-1.9)
Female 19–71+	69.3 (66.5-72.1)	62.1 (59.2-65.1)*	54.1 (51.1-57.1)*	45.4 (42.5-48.4)*	36.3 (33.4-39.2)*	1.2 (0.6-1.8)	1.5 (0.8-2.1)	1.8 (1.0-2.6)	2.2 (1.4-3.1)	2.8 (1.8-3.8)
Adult 19–71+	69.6 (67.4-71.8)	63.7 (61.4-66.0)*	57.2 (54.8-59.6)*	50.1 (47.7-52.6)*	42.6 (40.2-45.0)*	0.9 (0.4-1.4)	1.0 (0.5-1.6)	1.3 (0.6-1.9)	1.6 (0.9-2.3)	2.0 (1.1-2.8)

*95% confidence intervals of percentages from those below/above recommended and modeled replacement do not overlap

[a]EAR, estimated average requirement: A nutrient intake value that is estimated to meet the requirement of half the healthy individuals in a group

[b]AI, adequate intake, is a recommended average daily nutrient intake level assumed to be adequate based on experimentally derived intake levels or approximations of observed mean nutrient intake by a group (or groups) of apparently healthy people

[c]One ounce equivalent (eq) per Protein Foods Group is 0.5 oz nuts and 1 oz meat (20, p. 49). Therefore, we modeled the replacement of 1, 2, 3, and 4 oz eqs meat with 0.5, 1, 1.5, and 2 oz walnuts.

Supplemental Table S3: Percentage of individuals with nutrient intake levels above the AI[a] at current intake (usual diet) and modeled intake when replacing meat with equivalent[b] amount of walnuts

Age-gender group	**Potassium** % above AI (95% confidence interval)				
	Current	Replacement 1 oz eq	Replacement 2 oz eq	Replacement 3 oz eq	Replacement 4 oz eq
Male 4–8	31.7 (26.4-37.0)	30.4 (25.2-35.5)	29.0 (23.9-34.0)	27.9 (22.8-32.9)	26.6 (21.6-31.6)
Male 9–13	29.5 (24.9-34.0)	28.4 (24.0-32.9)	27.4 (23.0-31.8)	26.3 (21.9-30.7)	25.1 (20.7-29.5)
Male 14–18	20.4 (15.8-25.1)	19.7 (15.3-24.2)	19.0 (14.6-23.5)	18.2 (13.9-22.6)	17.6 (13.4-21.8)
Male 4–18	26.7 (24.0-29.5)	25.7 (23.0-28.5)	24.7 (22.0-27.4)	23.7 (21.1-26.4)	22.7 (20.1-25.4)
Female 4–8	21.8 (18.1-25.4)	20.5 (16.9-24.2)	19.4 (15.8-23.0)	18.3 (14.8-21.9)	17.2 (13.7-20.7)
Female 9–13	27.8 (23.4-32.2)	26.6 (22.2-30.9)	25.2 (20.9-29.4)	23.9 (19.7-28.1)	22.8 (18.7-26.8)
Female 14–18	21.8 (17.1-26.4)	20.8 (16.3-25.4)	19.8 (15.2-24.3)	18.7 (14.3-23.2)	17.8 (13.5-22.1)
Female 4–18	23.9 (21.5-26.4)	22.8 (20.4-25.2)	21.6 (19.2-24.0)	20.4 (18.1-22.8)	19.4 (17.1-21.7)
Child 4–18	25.4 (23.3-27.5)	24.3 (22.3-26.4)	23.2 (21.2-25.3)	22.2 (20.1-24.2)	21.1 (19.1-23.2)*
Male 19–50	22.9 (20.3-25.6)	22.3 (19.6-24.9)	21.6 (19.0-24.3)	21.0 (18.4-23.6)	20.5 (17.9-23.0)
Female 19–50	22.8 (19.6-26.1)	21.9 (18.7-25.1)	21.0 (17.8-24.2)	20.2 (17.1-23.3)	19.3 (16.2-22.4)
Adult 19–50	22.9 (20.6-25.2)	22.1 (19.8-24.4)	21.4 (19.1-23.6)	20.6 (18.4-22.9)	19.9 (17.8-22.1)
Male 51–70	25.3 (21.8-28.8)	24.6 (21.1-28.1)	23.9 (20.5-27.4)	23.4 (20.0-26.7)	22.7 (19.3-26.0)
Female 51–70	23.9 (19.5-28.2)	22.8 (18.5-27.2)	21.8 (17.5-26.0)	20.9 (16.7-25.0)	20.0 (16.0-24.0)
Adult 51–70	24.6 (22.3-26.9)	23.7 (21.5-26.0)	22.9 (20.6-25.1)	22.1 (19.9-24.4)	21.4 (19.2-23.5)
Adult 71+	17.3 (14.5-20.1)	16.3 (13.6-19.1)	15.5 (12.9-18.2)	14.7 (12.2-17.3)	14.0 (11.5-16.5)
Male 19–71+	22.9 (20.9-25.0)	22.2 (20.2-24.3)	21.6 (19.6-23.6)	21.0 (19.0-23.0)	20.4 (18.5-22.4)
Female 19–71+	22.7 (20.5-24.9)	21.7 (19.6-23.8)	20.7 (18.6-22.9)	19.9 (17.8-22.0)	19.0 (17.0-21.0)
Adult 19–71+	22.8 (21.1-24.5)	22.0 (20.3-23.7)	21.2 (19.5-22.9)	20.5 (18.8-22.1)	19.7 (18.1-21.4)

*95% confidence intervals of percentages from those below/above recommended and modeled replacement do not overlap

[a]AI, adequate intake, is a recommended average daily nutrient intake level assumed to be adequate based on experimentally derived intake levels or approximations of observed mean nutrient intake by a group (or groups) of apparently healthy people
[b]One ounce equivalent (eq) per Protein Foods Group is 0.5 oz nuts and 1 oz meat (20, p. 49). Therefore, we modeled the replacement of 1, 2, 3, and 4 oz eqs meat with 0.5, 1, 1.5, and 2 oz walnuts.

Supplemental Table S4: Percentage of individuals with nutrient intakes below the EAR[a] at current intake (usual diet) and modeled intake when replacing meat with equivalent[b] amount of walnuts

Age-gender group	**Copper** % below EAR (95% confidence interval)					**Zinc** % below EAR (95% confidence interval)				
	Current	Replacement 1 oz eq	Replacement 2 oz eq	Replacement 3 oz eq	Replacement 4 oz eq	Current	Replacement 1 oz eq	Replacement 2 oz eq	Replacement 3 oz eq	Replacement 4 oz eq
Male 4–8	1.0 (0.5-1.5)	0.0 (0.0-0.0)*	0.0 (0.0-0.0)*	0.0 (0.0-0.0)*	0.0 (0.0-0.0)*	1.6 (0.7-2.4)	4.3 (2.5-6.1)*	9.5 (6.1-12.8)*	17.8 (12.9-22.7)*	28.5 (22.0-35.0)*
Male 9–13	11.5 (7.5-15.6)	0.9 (0.3-1.5)*	0.0 (0.0-0.0)*	0.0 (0.0-0.0)*	0.0 (0.0-0.0)*	17.9 (13.7-22.1)	26.3 (21.2-31.4)	36.1 (30.2-42.0)*	45.3 (39.3-51.2)*	53.4 (47.9-58.9)*
Male 14–18	26.6 (22.6-30.6)	6.4 (4.5-8.2)*	0.5 (0.2-0.8)*	0.0 (0.0-0.0)*	0.0 (0.0-0.0)*	32.8 (27.5-38.1)	40.6 (35.3-45.8)	47.8 (42.6-53.1)*	54.2 (49.2-59.2)*	59.9 (54.9-64.8)*
Male 4–18	14.0 (11.7-16.3)	2.7 (1.9-3.5)*	0.2 (0.1-0.3)*	0.0 (0.0-0.0)*	0.0 (0.0-0.0)*	18.6 (15.7-21.4)	25.0 (22.0-28.0)*	32.4 (29.2-35.7)*	40.3 (37.0-43.6)*	48.3 (44.9-51.7)*
Female 4–8	1.2 (0.6-1.8)	0.0 (0.0-0.0)*	0.0 (0.0-0.0)*	0.0 (0.0-0.0)*	0.0 (0.0-0.0)*	3.8 (2.3-5.2)	8.9 (6.3-11.4)*	17.8 (13.9-21.6)*	30.0 (25.1-34.9)*	44.3 (38.4-50.2)*
Female 9–13	12.7 (8.8-16.6)	0.9 (0.5-1.4)*	0.0 (0.0-0.0)*	0.0 (0.0-0.0)*	0.0 (0.0-0.0)*	28.5 (23.4-33.7)	39.1 (33.6-44.7)	50.3 (44.5-56.2)*	59.9 (54.5-65.4)*	68.2 (62.9-73.4)*
Female 14–18	38.8 (33.0-44.6)	9.0 (6.1-12.0)*	0.6 (0.1-1.1)*	0.0 (0.0-0.0)*	0.0 (0.0-0.0)*	41.8 (36.1-47.6)	52.7 (47.0-58.3)	62.2 (56.7-67.6)*	70.0 (64.9-75.2)*	76.4 (71.8-80.9)*
Female 4–18	18.5 (15.4-21.7)	3.5 (2.4-4.7)*	0.2 (0.1-0.4)*	0.0 (0.0-0.0)*	0.0 (0.0-0.0)*	26.0 (23.2-28.8)	35.1 (32.0-38.1)*	45.0 (41.6-48.4)*	54.8 (51.3-58.2)*	64.1 (60.5-67.7)*
Child 4–18	16.2 (14.0-18.3)	3.1 (2.3-3.9)*	0.2 (0.1-0.3)*	0.0 (0.0-0.0)*	0.0 (0.0-0.0)*	22.1 (20.0-24.2)	29.8 (27.5-32.1)*	38.4 (35.9-40.9)*	47.2 (44.7-49.7)*	55.8 (53.2-58.5)*
Male 19–50	14.1 (11.6-16.6)	2.5 (1.7-3.3)*	0.2 (0.1-0.3)*	0.0 (0.0-0.0)*	0.0 (0.0-0.0)*	32.6 (29.5-35.6)	39.4 (36.3-42.5)*	46.0 (43.0-49.0)*	52.0 (48.9-55.0)*	57.0 (54.2-59.9)*
Female 19–50	25.0 (21.7-28.3)	4.6 (3.2-5.9)*	0.2 (0.0-0.3)*	0.0 (0.0-0.0)*	0.0 (0.0-0.0)*	30.8 (27.2-34.5)	41.2 (37.0-45.3)*	51.6 (47.2-56.0)*	60.9 (56.6-65.2)*	68.7 (64.7-72.7)*
Adult 19–50	19.1 (16.5-21.6)	3.4 (2.5-4.4)*	0.2 (0.0-0.3)*	0.0 (0.0-0.0)*	0.0 (0.0-0.0)*	31.8 (29.3-34.3)	40.2 (37.6-42.9)*	48.5 (45.9-51.2)*	56.0 (53.4-58.7)*	62.3 (59.9-64.8)*
Male 51–70	12.7 (10.5-14.8)	1.8 (1.1-2.5)*	0.1 (0.0-0.1)*	0.0 (0.0-0.0)*	0.0 (0.0-0.0)*	35.4 (31.9-39.0)	43.2 (39.1-47.3)*	50.2 (45.9-54.5)*	56.3 (51.8-60.7)*	61.2 (57.0-65.4)*
Female 51–70	25.6 (21.6-29.7)	4.2 (2.7-5.6)*	0.1 (0.0-0.3)*	0.0 (0.0-0.0)*	0.0 (0.0-0.0)*	35.2 (31.2-39.2)	46.8 (42.3-51.2)*	57.5 (52.6-62.4)*	66.5 (62.0-71.1)*	74.1 (69.9-78.3)*
Adult 51–70	18.9 (16.2-21.7)	3.0 (2.0-3.9)*	0.1 (0.0-0.2)*	0.0 (0.0-0.0)*	0.0 (0.0-0.0)*	35.3 (32.0-38.6)	44.9 (41.3-48.6)*	53.7 (49.9-57.5)*	61.3 (57.6-64.9)*	67.4 (64.1-70.8)*
Adult 71+	18.7 (11.1-26.3)	2.1 (0.1-4.1)*	0.0 (0.1-0.1)*	0.0 (0.0-0.0)*	0.0 (0.0-0.0)*	44.9 (38.7-51.2)	55.6 (49.6-61.6)	65.1 (59.6-70.7)*	73.3 (68.6-77.9)*	79.5 (75.5-83.4)*
Male 19–71+	13.7 (12.0-15.5)	2.2 (1.6-2.8)*	0.1 (0.0-0.2)*	0.0 (0.0-0.0)*	0.0 (0.0-0.0)*	35.0 (32.8-37.2)	42.3 (40.0-44.5)*	49.1 (46.8-51.3)*	55.1 (52.8-57.3)*	60.0 (57.9-62.1)*
Female 19–71+	24.8 (21.6-28.0)	4.2 (2.9-5.5)*	0.2 (0.0-0.3)*	0.0 (0.0-0.0)*	0.0 (0.0-0.0)*	33.3 (30.8-35.9)	44.4 (41.5-47.2)*	55.0 (51.9-58.1)*	64.3 (61.5-67.2)*	72.0 (69.5-74.5)*
Adult 19–71+	19.0	3.2	0.1	0.0	0.0	34.2	43.3	51.9	59.4	65.7

	(16.8-21.2)	(2.3-4.0)*	(0.0-0.2)*	(0.0-0.0)*	(0.0-0.0)*	(32.5-36.0)	(41.3-45.3)*	(49.8-54.0)*	(57.4-61.5)*	(63.9-67.5)*

*95% confidence intervals of percentages from those below/above recommended and modeled replacement do not overlap
[a]EAR, estimated average requirement: A nutrient intake value that is estimated to meet the requirement of half the healthy individuals in a group
[b]One ounce equivalent (eq) per Protein Foods Group is 0.5 oz nuts and 1 oz meat (20, p. 49). Therefore, we modeled the replacement of 1, 2, 3, and 4 oz eqs meat with 0.5, 1, 1.5, and 2 oz walnuts.

Supplemental Table S5: AMDR[a] of protein intake (protein as % energy) at current intake and modeled intake when replacing meat with equivalent[b] amount of walnuts

**Age-gender group**	**Protein as % of Energy Intake** % within AMDR[a] (95% confidence interval)				
	Current	Replacement 1 oz eq	Replacement 2 oz eq	Replacement 3 oz eq	Replacement 4 oz eq
Male 4–8	96.9 (95.6-98.3)	89.2 (86.2-92.1)*	71.7 (68.0-75.4)*	48.9 (45.2-52.6)*	31.2 (27.7-34.7)*
Male 9–13	97.1 (96.1-98.0)	90.8 (88.8-92.8)*	75.7 (71.5-79.8)*	54.7 (48.3-61.1)*	38.5 (32.1-44.9)*
Male 14–18	98.1 (97.2-99.0)	94.0 (91.8-96.2)*	82.5 (78.2-86.9)*	64.6 (58.7-70.5)*	49.0 (42.8-55.2)*
Male 4–18	97.4 (96.5-98.3)	91.5 (89.6-93.4)*	77.0 (74.0-80.1)*	56.7 (53.0-60.3)*	40.2 (36.4-44.0)*
Female 4–8	94.4 (91.6-97.1)	80.2 (75.7-84.8)*	56.3 (51.3-61.3)*	32.7 (28.5-36.9)*	17.4 (14.5-20.2)*
Female 9–13	94.2 (92.3-96.2)	82.1 (78.2-86.1)*	60.3 (54.9-65.7)*	38.0 (32.3-43.8)*	23.0 (18.4-27.6)*
Female 14–18	96.9 (95.3-98.5)	88.4 (84.8-92.0)*	67.9 (62.7-73.0)*	43.4 (37.8-49.0)*	26.2 (20.9-31.4)*
Female 4–18	95.2 (93.4-97.0)	83.8 (80.6-87.0)*	61.8 (58.2-65.4)*	38.4 (35.1-41.6)*	22.5 (19.7-25.3)*
Child 4–18	96.4 (95.1-97.6)	87.8 (85.5-90.1)*	69.8 (67.1-72.5)*	48.0 (45.1-50.8)*	31.8 (28.8-34.7)*
Male 19–50	98.6 (98.1-99.2)	96.2 (95.0-97.4)*	89.0 (86.9-91.1)*	75.6 (72.5-78.7)*	61.4 (57.6-65.1)*
Female 19–50	98.0 (97.2-98.9)	92.2 (90.0-94.4)*	76.7 (72.9-80.4)*	54.1 (49.3-58.9)*	35.2 (30.7-39.7)*
Adult 19–50	98.4 (97.8-99.0)	94.4 (92.9-95.9)*	83.4 (80.9-85.9)*	65.8 (62.4-69.3)*	49.5 (45.9-53.1)*
Male 51–70	98.9 (98.4-99.5)	96.8 (95.4-98.1)*	89.7 (86.4-93.0)*	75.2 (70.6-79.8)*	59.9 (55.4-64.4)*
Female 51–70	98.5 (97.4-99.6)	93.2 (90.3-96.1)*	77.3 (72.0-82.6)*	52.9 (47.2-58.5)*	32.8 (28.2-37.5)*
Adult 51–70	98.7 (97.9-99.5)	95.0 (93.0-97.0)*	83.7 (79.6-87.8)*	64.4 (59.7-69.1)*	46.8 (42.6-51.0)*
Adult 71+	98.7 (97.8-99.6)	93.4 (90.5-96.3)*	78.2 (72.8-83.7)*	55.3 (48.9-61.6)*	34.2 (28.4-39.9)*
Male 19–71+	98.8 (98.3-99.3)	96.4 (95.2-97.5)*	88.9 (86.6-91.2)*	74.6 (71.7-77.6)*	59.6 (56.5-62.7)*
Female 19–71+	98.2 (97.4-99.1)	92.4 (90.1-94.7)*	76.3 (72.5-80.1)*	52.7 (48.6-56.8)*	33.1 (29.6-36.6)*

Adult 19–71+	98.5 (97.9-99.1)	94.5 (92.9-96.0)*	82.9 (80.3-85.6)*	64.3 (61.3-67.3)*	47.1 (44.2-49.9)*

*95% confidence intervals of percentages from those below/above recommended and modeled replacement do not overlap

[a] The Acceptable Macronutrient Distribution Range (AMDR) (10%–35% of calories as protein)

[b]One ounce equivalent (eq) per Protein Foods Group is 0.5 oz nuts and 1 oz meat (20, p. 49). Therefore, we modeled the replacement of 1, 2, 3, and 4 oz eqs meat with 0.5, 1, 1.5, and 2 oz walnuts.

Supplemental Table S6: Saturated fat intake in grams and percentage of individuals with cholesterol intake levels above 300 mg at current intake (usual diet) and modeled for replacing meat with an equivalent[a] amount of walnuts

**Age-gender group**	**Saturated Fat** Grams (95% confidence interval)					**Cholesterol** % above 300 mg (95% confidence interval)				
	Current	Replacement 1 oz eq	Replacement 2 oz eq	Replacement 3 oz eq	Replacement 4 oz eq	Current	Replacement 1 oz eq	Replacement 2 oz eq	Replacement 3 oz eq	Replacement 4 oz eq
Male 4–8	24.2 (22.6-25.8)	24.3 (22.7-25.9)	24.4 (22.8-26.0)	24.5 (22.9-26.1)	24.6 (23.0-26.2)	18.9 (16.0-21.7)	15.3 (12.7-18.0)	12.2 (9.7-14.7)*	9.6 (7.2-12.0)*	7.3 (5.2-9.3)*
Male 9–13	27.4 (25.9-28.8)	27.5 (26.0-28.9)	27.6 (26.1-29.0)	27.7 (26.2-29.1)	27.8 (26.3-29.3)	28.8 (24.4-33.3)	24.2 (20.1-28.3)	20.6 (16.8-24.3)*	17.2 (13.9-20.5)*	14.4 (11.4-17.4)*
Male 14–18	30.1 (28.2-32.0)	30.2 (28.3-32.1)	30.3 (28.4-32.2)	30.4 (28.5-32.3)	30.5 (28.6-32.4)	38.2 (31.9-44.6)	33.1 (27.2-38.9)	29.0 (23.7-34.3)	25.3 (20.2-30.5)*	21.6 (16.8-26.4)*
Male 4–18	27.4 (26.4-28.5)	27.5 (26.5-28.6)	27.6 (26.6-28.7)	27.7 (26.7-28.8)	27.8 (26.8-28.9)	29.4 (26.4-32.4)	24.9 (22.1-27.6)	21.2 (18.6-23.8)*	18.0 (15.5-20.4)*	15.0 (12.7-17.2)*
Female 4–8	21.6 (20.4-22.9)	21.7 (20.5-23.0)	21.8 (20.6-23.1)	22.0 (20.7-23.2)	22.1 (20.8-23.3)	11.5 (8.6-14.3)	9.0 (6.3-11.6)	6.7 (4.4-8.9)	4.9 (3.0-6.9)*	3.5 (1.9-5.0)*
Female 9–13	25.0 (23.9-26.1)	25.1 (24.0-26.2)	25.2 (24.1-26.3)	25.3 (24.2-26.5)	25.4 (24.3-26.6)	19.3 (15.6-23.0)	15.4 (11.9-19.0)	12.5 (9.3-15.6)*	9.8 (7.2-12.5)*	7.8 (5.6-10.0)*
Female 14–18	23.4 (21.9-24.9)	23.5 (22.0-25.0)	23.6 (22.1-25.1)	23.7 (22.2-25.3)	23.8 (22.3-25.4)	19.6 (15.7-23.6)	16.1 (12.4-19.8)	12.8 (9.5-16.2)	10.5 (7.8-13.2)*	8.6 (6.1-11.0)*
Female 4–18	23.5 (22.6-24.3)	23.6 (22.7-24.4)	23.7 (22.8-24.5)	23.8 (22.9-24.6)	23.9 (23.0-24.7)	17.1 (14.7-19.6)	13.8 (11.4-16.1)	10.9 (8.8-13.0)*	8.6 (6.9-10.4)*	6.8 (5.3-8.3)*
Child 4–18	25.5 (24.8-26.3)	25.6 (24.9-26.4)	25.7 (25.0-26.5)	25.9 (25.1-26.6)	26.0 (25.2-26.7)	23.5 (21.3-25.8)	19.6 (17.4-21.7)	16.3 (14.3-18.3)*	13.5 (11.7-15.3)*	11.1 (9.5-12.7)*
Male 19–50	31.8 (30.6-33.1)	31.9 (30.7-33.2)	32.1 (30.8-33.3)	32.2 (30.9-33.4)	32.3 (31.0-33.5)	55.4 (52.2-58.6)	49.9 (46.6-53.2)	45.0 (41.9-48.1)*	40.8 (37.8-43.8)*	36.8 (33.8-39.8)*
Female 19–50	23.4 (22.5-24.3)	23.5 (22.6-24.4)	23.6 (22.7-24.5)	23.7 (22.8-24.6)	23.8 (22.9-24.7)	31.6 (28.3-35.0)	26.6 (23.5-29.8)	22.2 (19.3-25.2)*	18.8 (16.1-21.6)*	15.8 (13.2-18.4)*
Adult 19–50	28.0 (27.1-28.9)	28.1 (27.2-29.0)	28.2 (27.3-29.1)	28.3 (27.4-29.2)	28.4 (27.5-29.3)	44.6 (41.9-47.3)	39.3 (36.7-42.0)	34.7 (32.2-37.2)*	30.8 (28.4-33.2)*	27.3 (24.9-29.6)*
Male 51–70	29.8 (27.9-31.7)	29.9 (28.1-31.8)	30.0 (28.2-31.9)	30.1 (28.3-32.0)	30.3 (28.4-32.1)	55.3 (49.9-60.8)	49.6 (44.0-55.3)	44.5 (39.1-49.9)	40.0 (34.6-45.5)*	35.8 (30.4-41.1)*
Female 51–70	21.7 (19.9-23.6)	21.8 (20.0-23.7)	21.9 (20.1-23.8)	22.0 (20.2-23.9)	22.2 (20.3-24.0)	29.8 (24.9-34.7)	24.5 (20.2-28.9)	20.2 (16.1-24.4)*	16.8 (13.1-20.5)*	13.7 (10.4-17.1)*
Adult 51–70	25.9 (24.5-27.3)	26.0 (24.6-27.4)	26.1 (24.7-27.5)	26.2 (24.9-27.6)	26.3 (25.0-27.7)	43.0 (38.9-47.0)	37.5 (33.6-41.3)	32.8 (29.1-36.4)*	28.8 (25.3-32.2)*	25.1 (21.9-28.3)*
Adult 71+	22.4 (21.2-23.5)	22.5 (21.3-23.6)	22.6 (21.4-23.7)	22.7 (21.6-23.9)	22.8 (21.7-24.0)	30.4 (24.8-36.1)	25.1 (20.0-30.3)	20.8 (16.3-25.2)	17.2 (13.3-21.0)*	14.2 (10.8-17.7)*
Male 19–71+	30.7 (29.7-31.6)	30.8 (29.8-31.7)	30.9 (29.9-31.8)	31.0 (30.0-32.0)	31.1 (30.1-32.1)	54.0 (51.2-56.9)	48.5 (45.7-51.2)	43.5 (40.9-46.1)*	39.2 (36.7-41.6)*	35.1 (32.8-37.5)*

Female 19–71+	22.4 (21.7-23.2)	22.5 (21.8-23.3)	22.6 (21.9-23.4)	22.8 (22.0-23.5)	22.9 (22.1-23.6)	29.9 (26.9-32.8)	24.8 (22.1-27.5)	20.5 (18.0-23.0)*	17.1 (14.8-19.4)*	14.2 (12.1-16.3)*
Adult 19–71+	26.8 (26.1-27.4)	26.9 (26.2-27.5)	27.0 (26.3-27.6)	27.1 (26.4-27.7)	27.2 (26.6-27.8)	42.6 (40.3-44.9)	37.3 (35.1-39.4)*	32.6 (30.6-34.6)*	28.7 (26.9-30.6)*	25.2 (23.5-27.0)*

*95% confidence intervals of percentages from those below/above recommended and modeled replacement do not overlap
[a]One ounce equivalent (eq) per Protein Foods Group is 0.5 oz nuts and 1 oz meat (20, p. 49). Therefore, we modeled the replacement of 1, 2, 3, and 4 oz eqs meat with 0.5, 1, 1.5, and 2 oz walnuts.

Supplemental Table S7: Percentage of individuals with nutrient intake levels above the AI[a] at current intake (usual diet) and modeled at replacing meat with an equivalent[b] amount of walnuts

**Age-gender group**	**Omega-6** % above AI (95% confidence interval)					**Omega-3** % above AI (95% confidence interval)				
	Current	Replacement 1 oz eq	Replacement 2 oz eq	Replacement 3 oz eq	Replacement 4 oz eq	Current	Replacement 1 oz eq	Replacement 2 oz eq	Replacement 3 oz eq	Replacement 4 oz eq
Male 4–8	61.7 (56.3-67.1)	97.4 (96.1-98.7)*	100.0 (100.0-100.0)*	100.0 (100.0-100.0)*	100.0 (100.0-100.0)*	69.9 (64.6-75.2)	100.0 (100.0-100.0)*	100.0 (100.0-100.0)*	100.0 (100.0-100.0)*	100.0 (100.0-100.0)*
Male 9–13	60.7 (57.2-64.2)	92.7 (90.0-95.3)*	99.9 (99.7-100.0)*	100.0 (100.0-100.0)*	100.0 (100.0-100.0)*	57.9 (53.4-62.4)	100.0 (99.9-100.0)*	100.0 (100.0-100.0)*	100.0 (100.0-100.0)*	100.0 (100.0-100.0)*
Male 14–18	43.2 (37.0-49.4)	73.1 (67.6-78.5)*	96.1 (94.3-98.0)*	100.0 (99.9-100.0)*	100.0 (100.0-100.0)*	43.0 (36.5-49.5)	98.7 (98.0-99.5)*	100.0 (100.0-100.0)*	100.0 (100.0-100.0)*	100.0 (100.0-100.0)*
Male 4–18	54.4 (51.4-57.5)	86.8 (84.3-89.3)*	98.5 (97.8-99.2)*	100.0 (100.0-100.0)*	100.0 (100.0-100.0)*	55.9 (52.3-59.5)	99.5 (99.2-99.8)*	100.0 (100.0-100.0)*	100.0 (100.0-100.0)*	100.0 (100.0-100.0)*
Female 4–8	59.8 (54.6-64.9)	97.3 (96.0-98.7)*	100.0 (100.0-100.0)*	100.0 (100.0-100.0)*	100.0 (100.0-100.0)*	70.1 (65.1-75.1)	100.0 (100.0-100.0)*	100.0 (100.0-100.0)*	100.0 (100.0-100.0)*	100.0 (100.0-100.0)*
Female 9–13	74.7 (70.6-78.8)	98.9 (98.2-99.5)*	100.0 (100.0-100.0)*	100.0 (100.0-100.0)*	100.0 (100.0-100.0)*	72.6 (67.6-77.6)	100.0 (100.0-100.0)*	100.0 (100.0-100.0)*	100.0 (100.0-100.0)*	100.0 (100.0-100.0)*
Female 14–18	58.1 (51.9-64.3)	95.1 (93.2-97.0)*	100.0 (100.0-100.0)*	100.0 (100.0-100.0)*	100.0 (100.0-100.0)*	58.2 (51.7-64.6)	100.0 (100.0-100.0)*	100.0 (100.0-100.0)*	100.0 (100.0-100.0)*	100.0 (100.0-100.0)*
Female 4–18	64.5 (61.6-67.4)	97.1 (96.2-98.0)*	100.0 (100.0-100.0)*	100.0 (100.0-100.0)*	100.0 (100.0-100.0)*	66.8 (63.5-70.1)	100.0 (100.0-100.0)*	100.0 (100.0-100.0)*	100.0 (100.0-100.0)*	100.0 (100.0-100.0)*
Child 4–18	59.2 (57.1-61.3)	91.7 (90.2-93.1)*	99.2 (98.8-99.6)*	100.0 (100.0-100.0)*	100.0 (100.0-100.0)*	61.1 (58.4-63.8)	99.7 (99.6-99.9)*	100.0 (100.0-100.0)*	100.0 (100.0-100.0)*	100.0 (100.0-100.0)*
Male 19–50	48.6 (45.7-51.4)	75.5 (72.6-78.3)*	95.4 (94.1-96.7)*	99.9 (99.9-100.0)*	100.0 (100.0-100.0)*	56.6 (53.3-59.8)	99.5 (99.2-99.8)*	100.0 (100.0-100.0)*	100.0 (100.0-100.0)*	100.0 (100.0-100.0)*
Female 19–50	56.4 (52.6-60.2)	91.8 (89.6-93.9)*	99.9 (99.9-100.0)*	100.0 (100.0-100.0)*	100.0 (100.0-100.0)*	68.6 (65.1-72.1)	100.0 (100.0-100.0)*	100.0 (100.0-100.0)*	100.0 (100.0-100.0)*	100.0 (100.0-100.0)*
Adult 19–50	52.1 (49.6-54.6)	82.9 (80.8-84.9)*	97.5 (96.7-98.2)*	100.0 (99.9-100.0)*	100.0 (100.0-100.0)*	62.0 (59.4-64.7)	99.7 (99.5-99.9)*	100.0 (100.0-100.0)*	100.0 (100.0-100.0)*	100.0 (100.0-100.0)*
Male 51–70	56.2 (51.0-61.4)	88.3 (85.7-90.9)*	99.4 (99.1-99.8)*	100.0 (100.0-100.0)*	100.0 (100.0-100.0)*	51.9 (46.9-57.0)	99.6 (99.3-99.9)*	100.0 (100.0-100.0)*	100.0 (100.0-100.0)*	100.0 (100.0-100.0)*
Female 51–70	54.7 (49.8-59.6)	94.9 (93.2-96.7)*	100.0 (100.0-100.0)*	100.0 (100.0-100.0)*	100.0 (100.0-100.0)*	63.5 (58.2-68.9)	100.0 (100.0-100.0)*	100.0 (100.0-100.0)*	100.0 (100.0-100.0)*	100.0 (100.0-100.0)*
Adult 51–70	55.4 (51.6-59.3)	91.5 (89.6-93.4)*	99.7 (99.5-99.9)*	100.0 (100.0-100.0)*	100.0 (100.0-100.0)*	57.6 (53.6-61.5)	99.8 (99.6-100.0)*	100.0 (100.0-100.0)*	100.0 (100.0-100.0)*	100.0 (100.0-100.0)*
Adult 71+	45.9 (37.5-54.3)	88.6 (84.0-93.1)*	99.6 (99.3-100.0)*	100.0 (100.0-100.0)*	100.0 (100.0-100.0)*	54.3 (46.7-61.8)	99.9 (99.7-100.0)*	100.0 (100.0-100.0)*	100.0 (100.0-100.0)*	100.0 (100.0-100.0)*
Male 19–71+	50.1 (48.0-52.1)	79.8 (77.7-81.9)*	96.9 (96.1-97.8)*	100.0 (99.9-100.0)*	100.0 (100.0-100.0)*	53.7 (51.2-56.2)	99.5 (99.3-99.8)*	100.0 (100.0-100.0)*	100.0 (100.0-100.0)*	100.0 (100.0-100.0)*

Female 19–71+	55.1 (52.8-57.5)	93.1 (91.6-94.6)*	100.0 (99.9-100.0)*	100.0 (100.0-100.0)*	100.0 (100.0-100.0)*	66.8 (64.4-69.1)	100.0 (100.0-100.0)*	100.0 (100.0-100.0)*	100.0 (100.0-100.0)*	100.0 (100.0-100.0)*
Adult 19–71+	52.5 (50.8-54.2)	86.1 (84.6-87.6)*	98.4 (97.9-98.8)*	100.0 (100.0-100.0)*	100.0 (100.0-100.0)*	59.9 (57.9-61.8)	99.8 (99.6-99.9)*	100.0 (100.0-100.0)*	100.0 (100.0-100.0)*	100.0 (100.0-100.0)*

*95% confidence intervals of percentages from those below/above recommended and modeled replacement do not overlap

[a]AI, adequate intake, is a recommended average daily nutrient intake level assumed to be adequate based on experimentally derived intake levels or approximations of observed mean nutrient intake by a group (or groups) of apparently healthy people

[b]One ounce equivalent (eq) per Protein Foods Group is 0.5 oz nuts and 1 oz meat (20, p. 49). Therefore, we modeled the replacement of 1, 2, 3, and 4 oz eqs meat with 0.5, 1, 1.5, and 2 oz walnuts.
